# Pilot Implementation of a Primary Care Disease Management Concept for Venous Leg Ulceration: Results of a Mixed-Methods Process Evaluation

**DOI:** 10.3390/healthcare12242552

**Published:** 2024-12-18

**Authors:** Thomas Fleischhauer, Regina Poß-Doering, Nina Sander, Gunter Laux, Michel Wensing, Joachim Szecsenyi, Jonas D. Senft

**Affiliations:** Department of General Practice and Health Services Research, University Hospital Heidelberg, 69120 Heidelberg, Germany; thomas.fleischhauer@med.uni-heidelberg.de (T.F.); regina.poss-doering@med.uni-heidelberg.de (R.P.-D.); nina.sander@med.uni-heidelberg.de (N.S.); gunter.laux@med.uni-heidelberg.de (G.L.); michel.wensing@med.uni-heidelberg.de (M.W.); joachim.szecsenyi@med.uni-heidelberg.de (J.S.)

**Keywords:** disease management, leg ulcer, general practice, patient education, wound healing, case management

## Abstract

Background: Within the project “Ulcus Cruris Care”, a disease management intervention to improve general practice care for patients with venous leg ulcer was developed, comprising online teaching for practice teams, standardized patient education, and case management. Implementation of the intervention was piloted and evaluated via a process evaluation. This study aims to evaluate contentedness with the intervention, implementation effort, implementation determinants, intervention fidelity, and perceived intervention effects using a mixed-methods process evaluation. Methods: The mixed-methods process evaluation explored the views of general practitioners, medical assistants and patients regarding the intervention components. Data were collected through semi-structured telephone interviews and a survey questionnaire. Qualitative data were first analyzed inductively, followed by a deductive–inductive approach based on the Theoretical Domains Framework. Survey data were analyzed descriptively. Results: Participants (n = 21) reported a strong contentedness with the intervention, high intervention fidelity, low implementation effort, and a change in perception of compression therapy as the central treatment element. Healthcare professionals emphasized increased patient education and patient and family involvement. Patients reported feeling better informed and empowered to take an active role in their treatment, primarily due to increased knowledge and skills in compression therapy. As a result, they were more content with their care and reported positive experiences with wound healing since trial participation. Conclusions: The Ulcus Cruris Care intervention can lead to a noticeable change in knowledge and potentially influence practice teams’ approach to venous leg ulcer management, facilitating a significantly more frequent use of compression therapy in VLU care. A confirmatory evaluation of potential effects in a definitive RCT seems warranted.

## 1. Introduction

Venous leg ulcerations (VLUs) are a leading cause for chronic wounds of the lower extremities [1,2]. Though studies report heterogenous numbers [3,4,5], according to a recent systematic review and meta-analysis, approximately 1% of the population and 3% of people over 80 years of age suffer from a VLU [6]. Due to rising life expectancies, the prevalence of VLUs is predicted to further increase, challenging healthcare systems and burdening patients [7,8]. Management of chronic wounds is exerted by multiple actors such as general practitioners (GP), specialists, outpatient departments, nursing services, and certified wound managers. Currently, there is an ongoing shift of focus to local therapy, not least due to the development of modern wound dressings, although there is no firm evidence for added value of dressing materials [9,10]. In VLU management, compression therapy is the cornerstone of causal treatment, as it compensates for the underlying venous insufficiency hindering wound healing. Compression therapy facilitates venous return, reduces venous reflux, and promotes wound healing by reducing tissue inflammation [11]. It is confirmed as the most effective conservative treatment for VLU by several systematic reviews and has the potential of nearly halving healing duration when adequately applied [12,13,14,15,16]. Hence, its regular implementation is key for improving VLU care. Despite its proven effectiveness and status as the gold standard in VLU management, compression therapy is only applied in 30–40% of patients according to international care analyses [4,17,18,19,20]. As a consequence, patients may suffer chronification, morbidity, and considerable impairment of quality of life [21,22,23]. The reason for this care deficit is assumed to be multifactorial. First, there is insufficient knowledge among caregivers regarding compression therapy and its practical application [19,24,25,26,27]. Second, there are no standardized outpatient care concepts for chronic wound therapy, and GP practices experience increasing time pressure for consultation [28,29]. Third, patients tend to assume a passive and uninformed role during wound treatment, even though patient education is considered a key factor for therapy adherence and wound healing [30,31,32,33,34,35,36,37].

The Ulcus Cruris Care (UCC) project was established to develop a care concept supporting standardized evidence-based VLU treatment and patient education tailored for GP practices. For this purpose, a multifaceted implementation intervention was designed comprising online team training for GP practices, software support for case management, and educational material for patients. The intervention was piloted in 20 German GP practices [38]. To facilitate a deeper understanding of implementation processes and perceived outcomes and to enable valid interpretations [39], the pilot was accompanied by a mixed-methods process evaluation to assess contentedness with the intervention, estimated effort and determinants of its implementation, and perceived effects.

## 2. Materials and Methods

### 2.1. Study Design

For the observational cross-sectional mixed-methods process evaluation, an exploratory convergent parallel design with both simultaneous and sequential elements [40] was chosen. Qualitative semi-structured guide-based problem-centered interviews and a study-specific quantitative survey were used, complemented by a sociodemographic questionnaire. By incorporating quantitative and qualitative data in a synergistic manner, a mixed-methods approach allowed for a thorough evaluation of individuals’ perceptions on and experiences with the implementation of interventions or programs [39]. The reporting of this study is based on the COREQ checklist [41].

**Figure 1:**
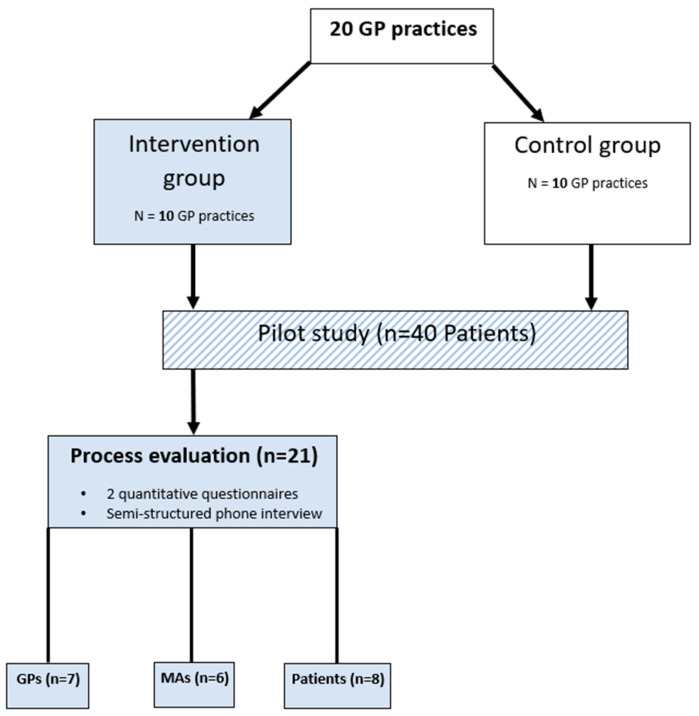
Study design of the process evaluation. GP = general practitioner. MA = medical assistant.

Figure 1 provides an illustration of the process of evaluation and study design.

### 2.2. Implementation Program

A multifaceted intervention program was developed to implement a disease management concept for outpatient treatment of VLU patients in GP practices. The intervention program comprised five main components: (1) online training and e-learning courses for GPs and medical assistants (MAs); (2) standard operating procedures (SOPs) for evidence-based VLU treatment; (3) software-supported case management; (4) involvement of MAs in case management, wound care, and patient education; and (5) print information and an e-learning course for patient education. The online training and e-learning had an approximate overall duration of 120 min and focused on the pathophysiology of VLU and chronic venous insufficiency, the effectiveness and practical aspects of compression therapy, local wound treatment, and patient education. Videos, graphics, illustrations, tables, and overviews were used for visualization of the content. A more detailed description of the intervention and its key features can be found in a previous publication [38].

All interventional components aimed to support an evidence-based and standardized disease management for VLU patients in GP practices and to foster patient education as well as active participation in VLU treatment. In the UCC pilot study, intervention components were rolled out and tested in 20 GP practices in the region of the federal state of Baden-Württemberg. During this phase and prior to implementing the intervention components in a prospective cluster-randomized controlled multicenter trial [42], a mixed-methods process evaluation explored the perceived contentedness, implementation effort, and effects of the program from participant perspectives.

### 2.3. Study Population

Target groups were GPs, MAs, and patients participating in the pilot study. A sample size of 10 GPs, 10 MAs, and 10 patients (n = 30) was aimed for.

### 2.4. Recruitment and Sampling

A pragmatic sampling strategy was pursued. GP practices had to include at least one patient in the pilot study to be eligible for participation in the process evaluation. GP practices within the German federal states of Baden-Wuerttemberg and Hesse that were actively using the CareCockpit (version 3.6.) were sent an information leaflet and informed consent form, which had to be signed and returned prior to enrollment. Patients within the intervention group could be enrolled by (participating, this word is optional) study practices after receiving information and giving written informed consent. In order to comply with data protection laws, no personal patient data were transmitted to the study center. Thus, patients had to initialize contact with the study center either themselves or via their GP practice in case of interest in participating. No expense allowances were paid to participants in the process evaluation.

### 2.5. Data Collection

Telephone interviews were conducted by a member of the study team (TF) using semi-structured interview guides. The experienced interviewer has a background in health sciences and health services research. Participants were interviewed either at home or at their workplace, and interviews were conducted either from the workplace or from the home office. Study-specific interview guides for the three participant groups (GPs, MAs, and patients) were designed by an interprofessional team (Health Services Research, General Practice) of researchers (TF, RPD, JDS) in an iterative process of assembling and appraising suitable questions. Questions were initially based on the pre-defined research questions, and wordings were adjusted after the first as well as after the first five interviews where considered necessary [43]. The interview guides focused on participants’ perceptions of the intervention components and were separated into thematic sections to explore (1) program fidelity, contentedness, and implementation effort; (2) the perceived effects of the interventions; and (3) contextual factors influencing implementation. Separate interview guides were developed for each target group. Interviews were conducted in the German language, digitally audio-recorded, and transcribed verbatim.

A non-validated, study-specific questionnaire was developed for capturing participants’ perceptions regarding the intervention components and perceived effects, as well as intervention fidelity and reach. The questionnaire comprised items regarding satisfaction, effort, and the perceived working mechanisms of (1) online training and e-learning courses for GPs and MAs; (2) standard operating procedures (SOPs) for evidence-based VLU treatment; (3) software-supported case management; (4) involvement of MA; and (5) print information and an e-learning course for patient education. Ratings used a 5-level Likert scale from 1 (totally disagree) to 5 (totally agree). A separate questionnaire was used to collect socio-demographic participant characteristics as well as practice characteristics. All data from the interviews and questionnaires were pseudonymized, digitally saved, and stored on secure servers at the Department of General Practice and Health Services Research of the University Hospital of Heidelberg.

### 2.6. Data Analysis

The qualitative data were analyzed in two phases and by two experienced members of the research team (TF, RPD). In phase one, an inductive framework analysis [44] was used to organize and manage interview transcripts in MAXQDA 2020 (Release 20.2.2). Enabling cross-case and cross-category comparison of qualitative data, framework analysis is a matrix-based systematic data-structuring approach that serves to identify central aspects and potential contrasts [45]. During this phase, inductive categories were formed referring to contentedness with and implementation effort of the implementation program, perceived effects of the intervention components, relevant contextual factors, and intervention fidelity. To achieve high intercoder congruence, coding of the qualitative data was discussed and, in case of divergence, approximated. Categories were discussed, adapted, and confirmed by the research team (TF, RPD, JDS) during regular consensus meetings.

Given the premise that implementing evidence-based interventions requires behavioral changes among providers and patients, the Theoretical Domains Framework (TDF) served as an analytical framework for identifying determinants of behavior changes in the deductive–inductive second phase of the qualitative data analysis [46,47]. To reflect the complexity of behavioral and organizational influencing factors, the TDF integrates 33 behavioral models and theories into a framework comprised of 14 theoretical domains. The application of the TDF facilitates the adaptation of intervention components to meet identified needs, barriers, and facilitators [46]. First, data were organized deductively by matching themes identified in phase 1 to corresponding TDF domains, and then inductively enriched with themes that arose directly from the data.

Descriptive analysis of sociodemographic and study-specific questionnaires was performed using Microsoft Excel software (Version 2301). Means, medians (MED), standard deviations (SD), maximum and minimum values, as well as percentiles and interquartile ranges (IQR) were calculated. For measuring intervention fidelity, absolute and relative frequencies of utilization, as reported by participants, were analyzed. The questions of the study-specific questionnaires were recoded from 1 to 5 (totally disagree = 1, somewhat disagree = 2, partially agree = 3, somewhat agree = 4, totally agree = 5). To enhance the support for interview statements through data triangulation, questionnaire items related to the same themes were consolidated into key statements.

## 3. Results

### 3.1. Participant and Practice Characteristics

Between July and December 2021, a total of n = 8 practices with n = 21 participants (7 GPs, 6 MAs and 8 patients) were interviewed and n = 20 (7 GPs, 6 MAs, and 7 patients) questionnaires were completed, corresponding to response rates of 75% and 71%. One practice was excluded since no patients were enrolled in this practice, and from one practice, consent to participate could not be obtained. Interview durations were between 08:31 and 54:27 min (mean: GPs = 20:16 min, MAs = 23:21 min, patients = 19:18 min; Overall mean = 20:58 min).

GPs were, on average, 56, MAs 50, and patients 70 years old. Three GPs (43%), six MAs (100%), and four patients (57%) were female. All GPs had a specialty in general practice, one of which had an additional specialty in internal medicine. Work experience in GP practices averaged 15.4 for GPs and 26 for MAs. Of the seven patients with a completed questionnaire, four (57%) were living alone and three (43%) together with a partner or relative at the time of data collection. GP practices were mostly rural (57%) and comprised two (29%) single practices, four (57%) joint practices, and one (14%) medical care center. On average, 4.3 physicians and 11.1 MAs, most of which had more than one additional qualification (83%), were employed per practice. The majority of practices were enrolled in GP-centered care (86%) or a physician network (57%) at the time of data collection. GP practices had an average of 1771.4 (±834.1) patients per quarter and 15.6 (±12.3) VLU patients per year.

### 3.2. Program Fidelity, Contentedness, and Implementation Effort

In the survey and during the interviews, participants rated their contentedness with the intervention very high and the effort of its implementation as rather low, as reflected by high intervention fidelity. In the interviews, GPs and MAs described being particularly content with the online training and e-learning course, as confirmed by the high contentedness ratings within the survey (GPs. 4.9 ± 0.4 and MAs 5 ± 0). The use of illustrative media was positively emphasized. Practice teams reported to have handed out information material to patients when deemed appropriate. The majority of patients received the paper-based material and were content with it: *“Very useful. There were things in there that you didn’t really know, right?”* (P08). Due to a lack of technical equipment, digital affinity, and support from relatives, only one patient used the digital information material.

The survey showed high satisfaction with the SOPs for GPs (4.6 ± 0.5) and MAs (4.7 ± 0.8). In the interviews, some described that they had them posted in their wound dressing room for easy access to the entire practice team. The SOPs were seen as useful for guiding clinical decisions in case of uncertainties, lacking experience, for delegation of tasks to MAs, or when a change in personnel occurred. They were also perceived as useful for comparison with and confirmation of one’s own approach or as reinsurance that nothing was forgotten.

GPs’ and MAs’ satisfaction with the software-supported wound documentation and monitoring was also high, although slightly lower compared to the other interventions (3.9 ± 0.7 and 4.3 ± 1.0). The standardized structure provided by the software, which ensured that all important parameters were included in the documentation, was appreciated by the interviewees. In the survey, patients indicated a perceived improvement in care since their participation in the pilot study (4.8 ± 0.4). Likewise, their satisfaction with care increased from 3.2 ± 1.6 before the study to 5 ± 0 after participation. Table 1 details participant ratings regarding intervention fidelity as well as perceived contentedness with and estimated effort of implementation of the interventions.

### 3.3. Perceived Effects of the Interventions

From GP practice teams’ perspectives as communicated during the interviews, the most useful and effective intervention was the online training plus additional e-learning. Patients saw most benefit in the combination of verbal education by the practice team plus written information material. The e-learning for patients was associated with several hurdles inhibiting usage, such as lacking technical equipment and cognitive capabilities or reservations about digital media. Due to a general issue of interoperability with applied practice software that could not be resolved regarding the newly developed software module, its usefulness was also seen as limited.

Based on the interviews, out of the 14 domains of the TDF, the domains most relevant to achieving behavior changes in VLU management, in particular concerning compression therapy, were “Behavioral regulation”, “Knowledge and skills” and “Social/professional role and identity”. First, GP practices refrain from prescribing and applying compression therapy regularly due to entrenched behavior patterns. Second, compression is underused due to provider deficits in knowledge and skills on compression therapy. Third, some GP practices have inhibitions to assume a role as the primary care provider for VLUs due to protracted treatment, frequent recurrences, and frustrating experiences with non-healing wounds.

**Figure 2:**
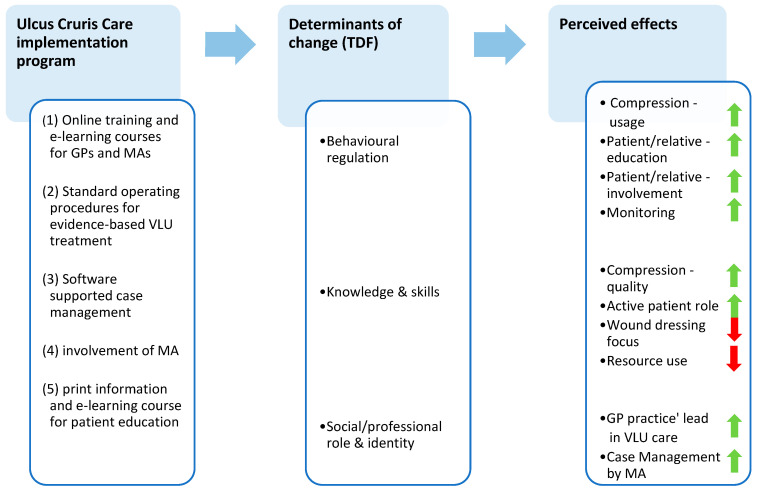
Theorizing analytical approach of the process evaluation. GP = general practitioner. MA = medical assistant. VLU = venous leg ulcer. 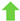 = increase. 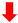 = decrease.

Participants’ perceptions on the interventions’ effects are reported based on these domains and are illustrated with translated quotes from the qualitative data, referenced with aliases (P = patient; GP = general practitioner; MA = medical assistant) and interview numbers. Figure 2 illustrates the theorizing analytical approach.

#### 3.3.1. Behavioral Regulation—Use of Compression Therapy and Patient Education

In the interviews, most GPs explicitly stated that compression therapy was not given sufficient importance prior to participation in the study. Also, several participants reported that the focus of treatment formerly used to be local wound treatment and the choice of wound dressing rather than compression therapy:

*“In the past, we only used the moist wound dressing, and that [the wound, author’s note] lasted for weeks and weeks, after I switched to the bandage and the compression, the wound healed much faster”.* (GP05)

Following the online training and e-learning, compression therapy was recognized as necessary for achieving wound healing in VLU by the interviewees, backed by the survey data of both GPs (5 ± 0) and MAs (4.8 ± 0.4). As reported by several interviewees and confirmed by the survey data, the choice of wound dressing is now seen as subordinate (GPs 2.4 ± 0.8 and MAs 2.7 ± 1.4). One GP stated that he was now treating patients successfully with compression therapy, even patients with particularly long pre-existing ulcers and a history of recurrences. Other GPs and MAs described that they developed a more rational behavior in wound dressing prescription and attached less importance to the choice of wound dressing (GPs 4.4 ± 1 and MAs 4.5 ± 0.5). In the interviews, some reported to have become more insistent with patients regarding compression therapy and to put more emphasis on patient education, particularly regarding compression therapy, as backed by the quantitative data (GPs 4.7 ± 0.5 and MAs 4.5 ± 0.5).

Patients also reported feeling better informed by their GP practice team in the interviews and survey (4.5 ± 1.4) and experienced improved support and treatment (4.6 ± 0.9). Within the interviews, patients stated to have developed a better understanding of their condition, compression therapy and its effectiveness, and better overall health behavior as indicated by the quantitative data (4.8 ± 0.5), with practice teams sharing this impression.

*“Raise your legs more often but also walk and not sit all day, so walk and run normally”.* (P03)/“*I have completely changed my eating habits”.* (P02)/“*It was always open, and you couldn’t do anything with it. Except wrap it. Of course, the leg would have to be wrapped every day”.* (P07)

The SOPs were used as instructions to guide clinical decisions, such as the choice of adequate compression device or wound dressing. According to the interviewees, they were seen as particularly useful for delegation of tasks to MAs or when care services become involved and for ensuring consistency, as well as for serving as a reference to prevent forgetting crucial steps. In some practices, the SOPs were made easily accessible to the entire practice team, e.g., by hanging them in their first aid cupboard or medical dressing room, where they could be reviewed regularly. Overall, the SOPs helped to implement evidence-based knowledge into practice and ensure that procedures were selected and carried out correctly, fostering standardization of VLU care, as indicated by the survey data (GPs 4.4 ± 1.1 and MAs 4.3 ± 0.5).

*“I think it’s very good that you have a clue as to what to do next so that you can perhaps get another thought or perhaps not forget anything if you call in a care service, for example, and so that you know how to do everything”. (MA04)/“They are now hanging in our first aid cupboard. They will also remain there, as they can be looked at again and again and remembered again and again”.* (GP03)

#### 3.3.2. Knowledge on and Skills in Compression Therapy and Patient Education

In the interviews, several participants reported an increase in refreshing and solidification of knowledge concerning the effectiveness and modalities of guideline-conform compression therapy following the interventions. The findings of the questionnaire revealed that, after the online training and e-learning, compression therapy has been recognized as the essential treatment element to achieve wound healing in VLU by GPs (5 ± 0), as well as by MAs and patients (4.83 ± 0.41). GPs and MAs reported feeling better informed on the application of compression therapy since participation in the study in both the interviews and survey questionnaires (4 ± 1.3 and 3.5 ± 1.6). For instance, MA06 said: *“So I think we put on the compression bandages better now”.* Some participants stated that they started questioning their application technique after they viewed the video on correct application of a two-layer compression bandage. *“In fact, the winding... because after I saw it like that, I asked myself whether it was always wound properly here or whether it was wound properly”.* (GP01). Other participants uttered that they made changes to their application technique afterwards, like using an underpadding.

According to both the interview and survey data, GPs (4.7 ± 0.5) and MAs (4.5 ± 0.5) felt better supported for patient education. Some practices instructed patients or the patients’ relatives on the correct application of a compression bandage, and two practices disseminated the knowledge gained from the webinar and e-learning to regional outpatient care services. Patients reported feeling better educated by the practice team regarding cause of disease as well as treatment. Many patients reported to have developed a better understanding of the disease and the necessity of compression therapy for wound healing in the interviews and surveys (4.5 ± 1) and changed their behavior correspondingly (4.75 ± 0.5): *“None of this happened because I was wearing the compression stockings”.* (P02)/*“It was always open, and you couldn’t do anything with it. Except wrap it. Of course, the leg would have to be wrapped every day”.* (P07). In addition, the interventions raised their awareness regarding general measures effective in wound healing, such as exercise and nutrition.

#### 3.3.3. GPs’ and MAs’ Social and Professional Role and Identity in VLU Care

As reported in the interviews, some GPs formerly referred patients regularly to specialists instead of treating them in their GP practice, e.g., because their treatment efforts with wound dressings did not yield success: *“I have sent on many patients with chronic ulcers, so in the past at least, and then of course treated them a lot with modern wound dressings, and partly very frustratingly”.* (GP02)

As shown by the survey data, both GPs (4.4 ± 0.8) and MAs (4.2 ± 1.2) perceived an increase in competence in VLU care through the interventions. In the interviews, these practices reported that they are now taking the lead in VLU care by retaining patients and treating them with compression therapy, with positive results:

*“These are also patients who have actually been treated for a long time and have now come back with a recurrence and … now it has made a difference, right. And before that [compression therapy, author’s note], with all kinds of expensive material, nothing, or always only short-term”.* (GP02)

Regarding the central role of MAs in VLU management, there was widespread agreement among the participants in the interviews that case management by MAs was to be advocated. Some argued that due to GPs’ time constraints, adequate care of VLU patients could not be realized without strong involvement of MAs in wound treatment and patient education. In most practices, one MA was mainly responsible for the care and management of VLU patients within the study and was *“given free reign”* (GP06). Some reported that they had always delegated wound care to the MAs, while others reported that they started delegating wound care visits and patient education to one mainly responsible MA during regular office hours.

Based on the interviews, providers and patients perceived a strengthening of the central role of MAs as case managers and an increased involvement in wound treatment and patient education. Several participants reported that the MAs often had better relations to the patients. Participants reported a number of benefits of giving MAs more responsibility and active involvement in the care and education of VLU patients, most notably the saving of resources due to an automatization and rationalization of processes:

*“Of course, it’s phantastic when they know what’s going on, the patients are automatically visited by the MAs and we only have to keep an eye on them or briefly check them or, in the case of complicated wound débridements or the like, join them, but otherwise the MAs take over quite autonomously, which of course speeds up the process intensively”.* (GP03)

This impression is confirmed by the survey data of both GPs (4.4 ± 1.1) and MAs (4.3 ± 0.5) suggesting an improved standardization of VLU care. Another advantage was seen *“in the external presentation of our practice that the whole team also shows medical expertise and competence”.* (GP03), which, according to the interviewees, was appreciated by patients (*“I found the practice competence, the doctor’s assistant or the wound dressing nurse who had the extra training, that was good”.* (P06)) and favored an upgrading of the profession. Concerning patient education and engagement, in the interviews, GPs and MAs stated an increased involvement of patients and their relatives in their treatment process, making use of their potential for self-care, as backed by MAs’ survey data (4.3 ± 0.5). For instance, two cases were reported where the spouse was instructed in compression therapy by the practice team and took over the daily application.

**Figure 3:**
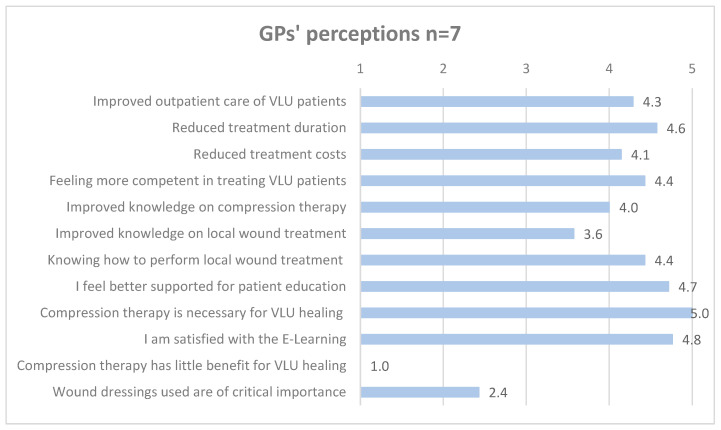
GPs’ mean ratings of perceived intervention effects. Likert scale ranging from 1 to 5 (totally disagree = 1, somewhat disagree = 2, partially agree = 3, somewhat agree = 4, totally agree = 5).

**Figure 4:**
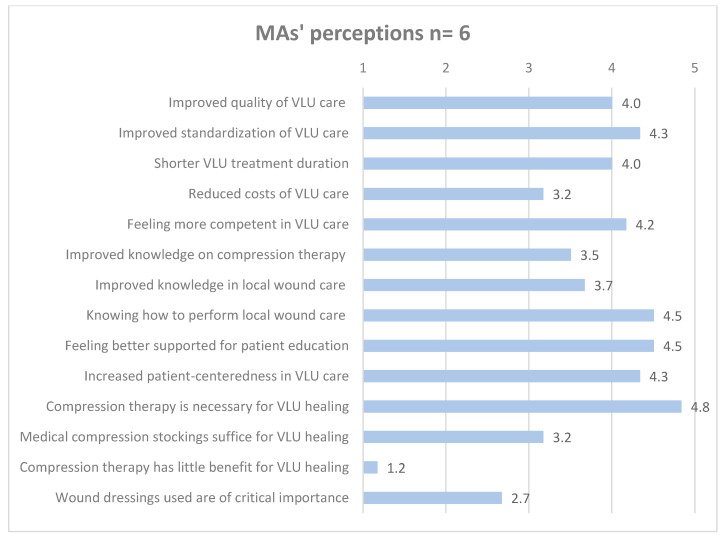
MAs’ mean ratings of perceived intervention effects. Likert scale ranging from 1 to 5 (totally disagree = 1, somewhat disagree = 2, partially agree = 3, somewhat agree = 4, totally agree = 5).

**Figure 5:**
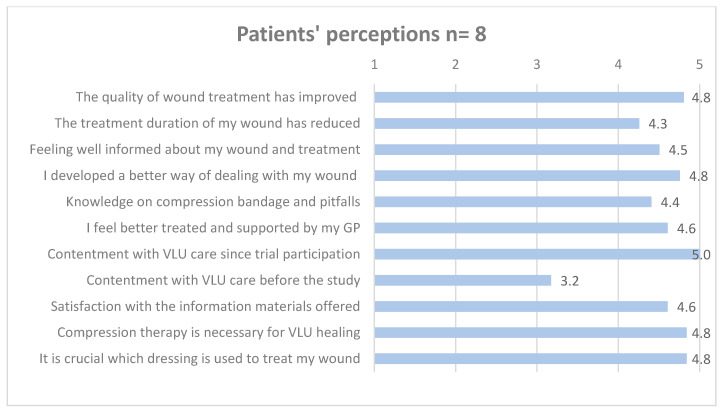
Patients’ means ratings of perceived effects of the interventions. Likert scale ranging from 1 to 5 (totally disagree = 1, somewhat disagree = 2, partially agree = 3, somewhat agree = 4, totally agree = 5).

Figure 3, Figure 4 and Figure 5 detail GPs’, MAs’, and patients’ mean ratings regarding perceived intervention effects.

### 3.4. Contextual Factors—Barriers and Enablers of Implementation

Several determinants of implementation were identified through the interviews. On an individual provider and patient level, the TDF domains “Beliefs in capabilities”, “Beliefs about consequences”, “Intentions”, “Goals”, “Emotions”, and “Social/professional role and identity” were identified as factors mediating the implementation of the UCC intervention (see Appendix A). However, for pragmatic reasons, only the most relevant domains, “Environmental context and resources” and “Social influences”, are depicted, as those factors were explicitly mentioned by most participants.

#### 3.4.1. Environmental Context and Resources

The most mentioned environmental context factor affecting the implementation of the interventions was resources. These comprise time, a particularly critical resource during the COVID-19 pandemic that was rampant at the time of the surveys, personnel, and money. According to the interviewees, VLU care was very time- and resource-consuming, with the compensation of expenses by health insurers being viewed as unsatisfactory:

*“… such an effort is often unavoidable…really time-consuming and yielding practically nothing, so in this lump-sum remuneration you actually have nothing left, no. So—time-consuming and “costs only”.* (GP04)

#### 3.4.2. Social Influences

The social network was mentioned as an important context factor influencing patient behavior and adherence. In some cases, the spouse or *“second doctor”* (P04) was in charge of daily application of the compression bandage. Also, the way patients are dealt with plays a crucial role in how patients perceive their treatment progress, influencing their motivation to *“stay on track”* (GP01).

In this context, one GP stated that his MA was more austere and stringent in motivating patients, whereas he often indulged the patients:

*“I tend to turn a blind eye, where the MAs try to motivate them so that the sloppiness doesn’t take hold too strongly…If we have already given up and can no longer motivate, then the MA is often the better contact person”.* (GP06)

While there was general support for delegating wound care and patient education to MAs, some skepticism persisted with reference to their qualification and patient preferences. For instance, one patient expressed a preference for primary treatment and education from their GP, at least at the onset of treatment. Both providers and patients mentioned that some patients did not want to be actively involved in treatment and patient education at all, and prefer being told what to do by the doctor:

*“(…) the patients prefer the doctor to say so-and-so-so before they read the big thing. At least the two we had in the study. (…) so, they really want everything to be prescribed and the doctor says “this is how we do it”. (…) I don’t know if they want to know more”.* (MA06)

## 4. Discussion

This process evaluation incorporated a mixed-methods approach to explore GPs’, MAs’, and patients’ perceptions on a complex disease management intervention. The evaluation focused on intervention contentedness, implementation effort, implementation determinants, intervention fidelity, and perceived intervention effects.

Participants in this process evaluation reported a strong contentedness with and low implementation effort of all intervention components, reflected by high intervention fidelity. A fundamental change in knowledge and perception of the role of compression therapy within VLU treatment was noticed, resulting in a focus shift from local wound treatment via topical wound dressings to conservative causal treatment via compression therapy. This is also confirmed by the quantitative analysis of the pilot study, which showed a high usage rate of 95% regarding guideline-conform compression therapy in patients of the intervention group [38].

Since research indicates significant deficits in knowledge on and practical application of compression therapy among all healthcare professionals [17,19,24,27], the remarkably high use of compression therapy in the intervention group suggests that the educational interventions effectively address this deficit. Compression therapy is confirmed as the most effective conservative treatment for VLU by several systematic reviews and has the potential of nearly halving healing duration when adequately applied and exerting a pressure within a range of 40–60 mmHg [12,13,14,15,16]. Hence, its regular implementation is the key factor for improving VLU care.

Next to addressing provider knowledge, fostering patient education and active treatment involvement should be a further priority for improving adherence to compression treatment. Lacking knowledge about the efficacy of compression therapy as well as patient-specific (such as pain and discomfort) and socio-psychological (aesthetic, patience, etc.) problems with compression therapy were identified as determinants of patient adherence [30,34,48,49,50]. Several studies, including systematic reviews, have shown that educational interventions to improve patient knowledge and adherence can improve both wound-related as well as patient-reported outcomes [51,52,53,54,55]. The educational interventions within UCC promoted informed and active participation of patients and relatives in the treatment process. Statements from the interviews suggest that most patients experienced an increase in knowledge and health literacy after exposure to the interventions, which is also reflected by patients’ ratings of compression therapy as very important. To ensure that information is properly understood by patients, it needs to be tailored to their abilities and needs [56]. Standardized verbal patient education in line with written information seem to have a high impact on patient knowledge [25,57]. Research suggests that multidimensional approaches to improving patient adherence, as pursued in the UCC project, may have greater potential to improve the care and quality of life of affected patients and reduce health-related economic costs than advances in local wound care [31,32,54]. The results of this study show that the combination of standardized verbal education and written information is satisfactory to patients and providers and effectively enhances patient knowledge and adherence, as reflected by the high rate of guideline-conform compression treatment. Therefore, standardized patient education, as carried out within the UCC intervention, should be an integral part of outpatient VLU care.

In some GP practices, before participation within UCC, VLU patients were routinely referred to specialists rather than being treated in-house. According to some interviewees, this was due to insufficient knowledge of appropriate treatment methods, past negative treatment experiences, a lack of standards in VLU care, and inadequate financial compensation. However, findings of this process evaluation indicate that the interventions enabled the establishment of a standardized and structured approach to diagnostics, treatment, and patient education by adapting their organizational structures and processes by means of delegation, centralization, and standardization. Through the learning curve effect and diminishing marginal effort associated with increased practice, this fosters the rationalization and efficiency of care processes in terms of economics of scale. Even though our findings suggest that—after appropriate training—GP practices can take lead as the primary care provider in VLU care, for managing particularly challenging ulcers, multi-disciplinary protocols can be needed [58,59].

Given the increasingly scarce resources of GP practices and expected rise in prevalence of multimorbidity and chronic wounds, fostering competence of MAs in chronic wound care and active involvement into treatment seems necessary to ensure adequate care of VLU patients. This study showed that strengthening and expanding the role of MAs in VLU care was strongly advocated by all participants, including patients. The case management approach and SOPs established within the UCC project seem to support this shift in responsibilities effectively. In the light of research showing that trust in nurses promotes patient adherence [60], as well as the statements of some interviewees that in wound care the relationship between MAs and patients is often closer than that between GPs and patients, the MA-centered approach to patient education pursued by the UCC project seems appropriate and effective. Routine integration of MA-led standardized patient education therefore seems warranted. For successful implementation of the UCC intervention into routine care, a remuneration needs to be linked to the additional training for GPs and MAs. Within the pilot study, compensation was offered to practices to account for expenditures not covered by as insufficient perceived routine reimbursements. Such a remuneration could be specified in selective contracts with certain health insurances. The UCC intervention incorporates elements usually familiar to GP practices, including case management, disease management, and the qualification and delegation of tasks to MAs. Given this alignment with existing practices and concepts, implementation into routine care is likely to be both feasible and relatively low effort.

### Strengths and Limitations

Several approaches to standardize and improve VLU care have been developed and described in the recent years. Among others, these comprise multi-disciplinary care models, the ABC model, the HEIDI mnemonic, “leg clubs”, community nurse care, or lifestyle-orientation programs [3,52,61,62,63]. However, these approaches have a rather narrow focus as they usually address either providers, such as physicians and nurses, or patients, not both at once. To our knowledge, this is the first mixed-methods process evaluation accompanying a disease management approach for chronic wound care in German general practices with a holistic focus on both providers and patients. This methodological approach allowed for an in-depth analysis of the implementation process and helped to further support or contrast statements from the interviews [39]. However, some limitations have to be considered. The target sample size could not be reached, yet a high degree of thematic saturation was achieved. Non-validated study specific questionnaires with unclear psychometric properties were used. Thus, validity and reliability, ceiling effects, or discriminability could not be accounted for. Regarding contextual factors influencing implementation, only those highlighted by several participants were considered. More individual psychological factors such as intentions and emotions that were extracted from interview transcripts were not considered within the result presentation, as systematic measures of these factors were not applied, but they are listed in Appendix A.

Lastly, the effects of the interventions were presented rather superficially via indication of the TDF domain addressed by the intervention. A more detailed depiction of working mechanisms would require identifying specific behavior change techniques inherent to the interventions, of which there are 93 in total according to the BCTTv1 framework [64]. However, as the interpretation of interventions’ impact on psychological behavior change is prone to uncertainty if based on interviews alone, an aggregated presentation of effects on TDF determinants seemed more appropriate since, in addition, those 93 behavior change techniques can be mapped into the 14 TDF domains [65].

## 5. Conclusions

The UCC intervention has the potential to effectively influence practice teams’ approaches to VLU management by promoting standardized guideline-conform disease management and patient education. Participation of GP practices and patients in the UCC intervention is associated with a noticeable change in knowledge and treatment behavior, resulting in a significantly more frequent use of compression treatment in VLU care. Broader dissemination of the intervention seems appropriate for improving chronic wound care. A confirmatory evaluation of potential effects of the UCC intervention in a definitive RCT seems warranted.

## Figures and Tables

**Table 1:** Perspectives on interventions and intervention fidelity.

Intervention Component	Intervention Fidelity n (%) *	General Practitioner Rating Mean ** (SD)	Medical Assistent Rating Mean ** (SD)	Patient Rating Mean ** (SD)
E-Learning and Webinar for practice teams	10 (100)			-
Contentedness Implementation effort		4.8 (0.5) 2.3 (1.1)	4.9 (1.4) 2.7 (0.8)	
Standard Operating Procedures	7 (86)			-
Contentedness Implementation effort		4.6 (0.5) 2 (1.2)	4.7 (0.8) 2.5 (0.8)	
Software support	6 (77)			-
Contentedness Implementation effort		3.9 (0.7) 3 (1.0)	4.3 (1.0) 3.5 (0.8)	
Written patient information	7 (86)	-	-	
Contentedness Implementation effort				4.6 (0.9) 3.3 (1.3)
E-Learning for patients	1 (10)	-	-	
Contentedness Implementation effort				5 (0) 1.5 (0.7)

SD = standard deviation. * Absolute and relative frequencies of usage. ** Likert scale ranging from 1 to 5 (totally disagree = 1, somewhat disagree = 2, partially agree = 3, somewhat agree = 4, totally agree = 5).

## Data Availability

All data generated and analyzed in this study are stored on a secure server at the University Hospital Heidelberg, Germany, Department of General Practice and Health Services Research. Due to data protection and privacy regulations, data is not available publicly. De-identified sets of the data collected and analyzed during this study can be made available by the corresponding author on reasonable request.

## Abbreviations

C-RCT	Cluster-randomized controlled trial
CVI	Chronic venous insufficiency
GP	General practitioner
MA	Medical assistant
SOP	Standard Operating Procedure
UCC	Ulcus Cruris Care project
VLU	Venous leg ulceration

## Appendix A

- Selected interview quotes on relevant TDF domains

**Contextual Factors/Barriers and Enablers of Implementation**	**Quote/Statement**	**Related TDF Domain**
Seemingly, those practices that were not already competent at and had possibly insufficient knowledge on VLU treatment—and especially compression therapy—profited most from the interventions. Those who were already confident in their knowledge and skills saw less need for additional education via e-learning.	Pre-existing knowledge and skills in compression therapy could also be the reason for the ambiguous results in MAs’ assessment of whether they now feel more competent in compression therapy. *“Because I’ve always had such a fixed programme and it works very well, it’s less useful to me now, I have to say”.* (GP07)	- Beliefs about capabilities - Knowledge - skills
The need for regular visits to the practice and the tediousness of the treatment could discourage patients from staying on track, as, e.g., one patient stated that she did not like *“That you had to go to the doctor again and again”. (P01)*	*“That it was just too slow for me until it was closed. But they can’t help it, it’s simply the wound healing process, which sometimes doesn’t happen as quickly as you’d like, right? Patience is just not our strength”.* (P06)	- Beliefs about capabilities - Behavioral regulation - Emotions
The patients’ personal interest in healing decisively influences their behavior, with some patients seemingly not wanting to be healed at all, according to the interviewees:	*“We have had a patient for 20 years who also has wounds, who rubs them and manipulates the wounds just so that she can receive, let’s say, care and that someone gives her attention… I think they rather have them taken care of than let someone get the wound under control quickly”.* (MA04)	- Intentions - Goals - Emotions
One important factor of successful implementation was seen in the practice owner’s attitude towards the study.	*“Well, because the boss also attached importance to it. That we now go through with it and do it”.* (MA06)	- Intentions - Goals - Social/professional role & identity
The same accounts for GPs’ and MAs’ intrinsic motivation to improve patient care even if it is associated with higher expenditures.	*“I think the most important secret of success is that the MAs—the A) themselves are interested in improving the care and that they really get involved”.* (GP06)	- Intentions - Goals
An important context factor influencing patient behaviour is the existence of a social network, such as having children or grandchildren, a spouse, or a neighbor who havz an eye on their relative or neighbor.	*“So, we have a concrete case where we were messing around for a long time with nappies and nutrition and so on, and we gave something, this information, and then the children came into play and since then people look at it differently”.* (GP02)/“*This usually works out quite well, especially when relatives are on board”.* (MA06)	- Social influences
Not all patients are willing to abandon their independence, not even to their spouse.	*“What I don’t like sometimes is that my husband always thinks too much, then I say “let me do it”, then he says “no, sit down”, does my foot again, and I want to stay independent for once”.* (P03)	- Social influences
Concerning patient education via e-learning and written information for patients, several participants stressed the importance of accessible and layman-friendly language to avoid making information unnecessarily difficult to access, since patients may have different conditions.	*„You know because there are also many other people who perhaps understand even less […] and yes for them it [the information, author’s note] is then Bohemian villages”.* (P01)	- Knowledge - Skills
Furthermore, the way patients are dealt with may play a crucial role in how patients perceive their treatment progress, possibly influencing their motivation to *“stay on track” (GP01).*	*“I am a very positive person and I praise a lot. That means that when a patient arrives and I see a slight improvement, I simply praise it. And I don’t go into negative aspects so much, but I am someone who simply says: “oh, you did a nice job” and “it looks like you also did a compression at night”... just such positive support”.* (GP07)	- Social influences - Optimism
This may be a particularly important approach for patients with depressive symptoms. For example, one patient stated to not have read the patient information due to psychological problems at that time which affected her interest:	*“And there was nothing good all around me. I then had no interest in anything”.* (P02)	- Emotions - Social influences
While advantages were seen in the e-learning format which made the hurdles of participation more low-threshold—especially during the peak of the pandemic—some participants uttered their preference for live trainings, which, according to them, allowed for more personal contact and exchange.	Some participants questioned the effectiveness of an e-learning format for conveying the skill of correct application of a compression bandage in general, which according to them is something a workshop could be better suited for.	- Beliefs about consequences
Context factors influencing the uptake of the e-learning and software are the degree of digital affinity—which seems to highly correlate with age—as well as individual learning preferences concerning, e.g., the teaching format and methods.	*“Young people can do it, but I’m 61 now and I’ve really... I know there are people who are 70 and over who are still doing it, but so far I’ve done so well”.* (P06)	- Beliefs about capabilities - Knowledge - skills
Concerning the central role of MAs in VLU care, besides the GPs’ and MAs’ attitudes, the individual patient preferences and their socialization seem to play a crucial role to whether greater responsibilities for MAs are accepted. There are patients who might have reservations and prefer being primarily treated and educated by the GP, at least in the beginning of treatment, as was explicitly mentioned by one patient.	A more patriarchal doctor image (“*demigod in white”, S03*) was mentioned to possibly undermine this approach. *“Well, I would definitely say that the doctor should have an eye on it at least the first two or three times and then I would have the confidence that she can do it on her own, but in the beginning I would like the doctor to be there”.* (P05)	- Social/professional role & identity - Social influences
Factors influencing the use of the software for documentation and monitoring were, for example, the scope of documentation or (time) effort, functionality, susceptibility to errors, integrability in everyday practice, and interoperability with the practice software, as well as previous experience with the CareCockpit.	No quote	- Knowledge - Skills - Environmental context & resources
